# Global pattern and trends of colorectal cancer survival: a systematic review of population-based registration data

**DOI:** 10.20892/j.issn.2095-3941.2020.0634

**Published:** 2021-09-06

**Authors:** Yufei Jiang, Huiyun Yuan, Zhuoying Li, Xiaowei Ji, Qiuming Shen, Jiayi Tuo, Jinghao Bi, Honglan Li, Yongbing Xiang

**Affiliations:** 1 State Key Laboratory of Oncogene and Related Genes & Department of Epidemiology, Shanghai Cancer Institute, Renji Hospital, Shanghai Jiao Tong University School of Medicine, Shanghai 200032, China; 2 Renji Hospital, Shanghai Jiao Tong University School of Medicine, Shanghai 200127, China; 3 School of Public Health, Shanghai Jiao Tong University School of Medicine, Shanghai 200025, China

**Keywords:** Colorectal cancer, survival rate, prognosis, population-based study, cancer registration

## Abstract

This review will describe the global patterns and trends of colorectal cancer survival, using data from the population-based studies or cancer registration. We performed a systematic search of China National Knowledge Infrastructure (CNKI), Wanfang Data, PubMed, Web of Science, EMBASE, and SEER and collected all population-based survival studies of colorectal cancer (up to June 2020). Estimates of observed and relative survival rates of colorectal cancer by sex, period, and country were extracted from original studies to describe the temporal patterns and trends from the late 1990s to the early 21st century. Globally, 5-year observed survival rates were higher in Seoul, Republic of Korea (1993–1997; 56.8% and 54.3% for colon and rectum cancers, respectively), Zhejiang province (2005–2010; 52.9% for colon cancer), Tianjin (1991–1999; 52.5% for colon cancer), Shanghai (2002–2006; 50.0% for rectum cancer) of China, and in Japan (1993–1996, 59.6% for colorectal cancer). Five-year relative survival rates of colorectal cancer in the Republic of Korea (2010–2014), Queensland, Australia (2005–2012), and the USA (2005–2009) ranked at relatively higher positions compared to other countries. In general, colorectal cancer survival rates are improving over time worldwide. Sex disparities in survival rates were also observed in the colon, rectum, and colorectal cancers in most countries or regions. The poorest age-specific 5-year relative survival rate was observed in patients > 75 years of age. In conclusion, over the past 3 decades, colorectal cancer survival has gradually improved. Geographic variations, sex differences, and age gradients were also observed globally in colorectal cancer survival. Further studies are therefore warranted to investigate the prognostic factors of colorectal cancer.

## Introduction

Colorectal cancer (CRC) is one of the most commonly diagnosed cancers in the world. According to Global Cancer Statistics 2020^1^, CRC ranks the third with 10.0% of newly detected cases (6.0% for colon and 3.8% for rectum) and the second in terms of deaths with 9.4% (5.8% for colon and 3.4% for rectum cancers). More than half of the cases are occurring in developed countries. More specifically, the regions of the world with the highest Human Development Index (HDI) are displaying, for both sexes, an incidence and mortality of CRC, which are at least twice as high as low index regions^2^.

The increasing burden of CRC is currently presenting major challenges to the world healthcare systems. Research on CRC is therefore committed to studying this burden and locating medical resources in a more equitable way. Population-based cancer registries are an essential component of all cancer prevention and control plans, providing comprehensive and accurate information on the incidence, mortality, survival, and other factors related to cancer in the population^3^. Compared to population-based incidence and mortality reports, studies on cancer survival are investigated and reported less frequently and not always in a timely manner. Survival data of cancer might not be available for countries or regions that have systematically reported the data of cancer incidence or/and mortality. There are 3 different sources of survival data, which are extracted from clinical trials, hospital-based follow-up studies, and population-based cancer registries. These sources may differ in terms of their aims, definition of survival-time, methods of survival estimation, and applications. Survival data based on clinical trials are mainly adopted as common indicators to evaluate the effectiveness of cancer therapies with respect to overall survival (OS, defined as the date from randomization to death from any cause) and progression-free survival (PFS, defined as the date from randomization until progression or death from any cause). Hospital-based survival analysis that collects survival information of patients who have been hospitalized generally reflects the service’s capacity and treatment effects within a specific department^4^. For instance, a phase 2 randomized clinical trial published in *JAMA Oncology* adopted a 10-month PFS as the primary end point to assess whether maintenance therapy with single agent panitumumab was as effective as panitumumab plus fluorouracil and leucovorin in patients with RAS wild-type, unresectable metastatic colorectal adenocarcinoma. This study showed that in terms of the 10-month PFS, maintenance therapy with single agent panitumumab [59.9%, 95% confidence interval (CI): 51.5%–69.8%] was inferior to panitumumab plus fluorouracil-leucovorin (49.0%, 95% CI: 40.5%–59.4%)^5^. A hospital-based survival study conducted by Sun Yat-sen University Cancer Center (Guangzhou, China) determined the associations between the survival rates and different clinical characteristics of CRC patients. The 5-year survival percentage (equivalent to a 5-year observed survival rate (OSR) was 70% and 77% during the 1990s and 2000s, respectively^6^. These results were higher than the population-based OSR in other geographical areas of China. For example, in Shanghai, during the same period, the 5-year OSR was 48.8% (2002–2006)^7^.

In the present study, we therefore assessed the survival rates of population-based cancer registries, by extracting the relative survival and observed survival rates. Our aim was to describe global patterns and chronological changes of observed and relative survival rates in CRC patients in different populations and/or regions covering the period from the 1990s to the early 21st century.

## Materials and methods

### Search strategy and data extraction

**Figure 1:**
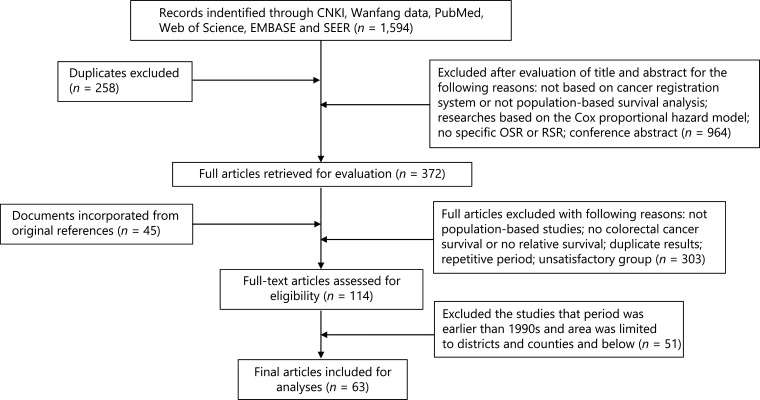
Study selection process.

A literature search of related studies up to June 2020 was conducted using the following databases: China National Knowledge Infrastructure (CNKI), Wanfang Data, PubMed, Web of Science, EMBASE, and SEER. The keywords included were: “colorectal cancer”, “colon cancer”, “rectum cancer”, “population-based survival studies”, “relative survival”, “observed survival”, and “cancer registry”. A total of 1,594 articles were found using the search strategy, including 258 duplicates. Two researchers independently collected the data according to the search criteria. A total of 372 articles were selected by browsing titles and abstracts. Studies were included if they: 1) provided relative survival rates (RSRs) or OSRs of CRC, 2) were not the net survival rates or survival rates considering the analysis of competing deaths, 3) were population-based or from cancer registries, and 4) were not assessing overlapping periods, incomplete, or unavailable articles. The final analysis included 63 studies after our screening, 5 of which were in Chinese and the remaining 58 were in English (**Figure 1**).

Since the International Classification of Diseases-10th Revision (ICD-10) included a further definition of malignant tumors (codes C00–C97) with respect to the 9th Revision (codes 140–208), the definition of CRC has improved. Anal cancer was classified as rectum cancer in ICD-9 (code 154) but was coded as C21, independently from rectum cancer (codes C19–C20) in ICD-10. However, some studies included in this review still combined cancer of the colon, rectum, and anus as CRC (codes C18–C21), which led to a slight inevitable defect of the present study.

The original data spanned from the 1960s to the early 21st century. Nevertheless, in consideration of the small significance that early data had for the present analysis, this study only displayed data from the 1990s.

### Statistical analysis

Estimates of RSRs and OSRs were extracted from the selected studies. RSR is defined as the ratio of the observed survival rate (where all causes of deaths are considered as events) to the expected survival rate (which is estimated from national population life tables stratified by sex, age, and calendar period) in the general population with the same distribution of key demographic factors (sex, age, calendar, period, and country)^4^. Note that RSR is a ratio rather than a rate; however, in practice it was often used as the term for “relative survival rate” and not “relative survival ratio”. RSR provides a measure of the excess mortality hazard experienced by cancer patients, irrespective of whether the excess mortality is directly or indirectly attributable to the cancer^8^. Also, RSR enables a direct comparison of survival rates between different populations or regions by eliminating the effects of age, sex, ethnicity, and calendar period on cancer survival to some extent. When comparing survival rates in different countries, times, or populations, the following was considered: selected indicator, additional comorbidities or variables, and whether death certificate only (DCO) cases or autopsy cases were excluded or not. In the present study, we displayed 1-, 3-, and 5-year relative and observed survival rates by sex and age. We distinguished between the age standardization RSRs and the overall RSRs in the outcomes to obtain better international comparisons^9^. In addition, some studies excluded patients < 15 years of age (or 20 years)^10–16^ and > 85 years of age during the analysis^11^, and a large number of studies excluded DCO cases or autopsy cases^10–15,17–39^. Endnote X9 and Excel (Microsoft, Redmond, WA, USA) and SPSS, version 22.0 (SPSS, Chicago, IL, USA) were used for literature management and data analyses.

## Results

### Global patterns and trends

**Table 1** shows population-based overall 1-, 3-, and 5-year OSRs of colon cancer, rectum cancer, and CRC in Asia^7,18,20,35,39–44^, America^18^, Europe^11–12,45–47^, and Africa^18,48–49^, respectively (all countries and regions involved were listed in **Supplementary Table S1**). Minor differences were detected in 1-year and 3-year OSRs between different regions, but in the 5-year OSRs, these differences were more apparent. For colon cancer, the highest 5-year OSR was observed in Seoul, the Republic of Korea (1993–1997) with 56.8%, followed by China, Zhejiang province (2005–2010), and Tianjin (1991–1999) with 52.9% and 52.5%, respectively. In Kampala, Uganda (1993–1997) and Bhopal, India (1991–1995), and Mumbai, India (1992–1999), the 5-year OSRs were poor, only 5.4%, 6.3%, and 27.4%, respectively. The OSRs of rectum cancer were similar to that of colon cancer in terms of patterns, although the rates in colon cancer tended to be slightly higher. For CRC, Japan (1993–1996) ranked the first with a 5-year OSR of 59.6%, whereas Kampala, Uganda (1993–1997) was the poorest with 6.6%.

**Table 1:** Population-based overall 1-, 3-, and 5-year observed survival rates of colon, rectum, and colorectal cancer during 1990–2015

Regions	Period	5-year OSRs (%)		
		1-year	3-year	5-year
**Colon**				
China				
Shanghai^7^	2002–2006	74.1	56.4	48.8
Zhejiang^40^	2005–2010	75.9	59.5	52.9
Tianjin^20^	1991–1999	69.5	55.6	52.5
Hong Kong^18^	1996–2001	74.8	57.2	48.9
Korea				
Seoul^18^	1993–1997	77.1	62.2	56.8
Busan^18^	1996–2001	74.7	56.9	49.0
Incheon^18^	1997–2001	69.6	54.7	48.7
Singapore^18^	1993–1997	70.3	51.0	41.8
Thailand				
Songkhla^18^	1990–1999	77.8	51.4	45.0
Lampang^18^	1990–2000	57.4	42.1	35.9
Chiang Mai^18^	1993–1997	61.5	39.7	29.3
Khon Kaen^18^	1993–1997	64.5	50.5	40.8
India				
Bhopal^18^	1991–1995	52.1	25.0	6.3
Mumbai^18^	1992–1999	52.5	35.1	27.4
Turkey				
Izmir^18^	1995–1997	73.8	56.3	44.9
Cuba^18^	1994–1995	58.7	39.5	35.3
Europe^45,46^	1990–1994	67.0	48.0	40.0
	1995–1999	69.5	51.3	42.8
Spain^12^	2000–2007	–	–	46.3
Uganda				
Kampala^18^	1993–1997	54.3	19.0	5.4
**Rectum**				
China				
Tianjin^20^	1991–1999	73.2	55.4	48.1
Hong Kong^18^	1996–2001	79.2	59.3	49.9
Shanghai^7^	2002–2006	79.8	60.9	51.7
Zhejiang^40^	2005–2010	77.4	57.0	48.7
Korea				
Seoul^18^	1993–1997	82.5	61.8	54.3
Busan^18^	1996–2001	82.5	59.7	49.6
Incheon^18^	1997–2001	77.7	58.7	49.6
Singapore^18^	1993–1997	73.5	52.5	42.7
Thailand				
Songkhla^18^	1990–1999	74.0	44.8	33.2
Lampang^18^	1990–2000	65.9	43.0	36.2
Chiang Mai^18^	1993–1997	69.7	36.9	27.9
Khon Kaen^18^	1993–1997	70.5	44.4	40.2
India				
Bhopal^18^	1991–1995	56.4	25.6	7.7
Mumbai^18^	1992–1999	59.7	37.2	28.6
Barshi^18^	1993–2000	46.4	16.9	11.2
Karunagappally^18^	1991–1997	76.3	49.4	26.5
Turkey				
Izmir^18^	1995–1997	82.9	55.9	44.1
Cuba^18^	1994–1995	70.9	47.0	42.1
Europe^45,46^	1990–1994	72.0	49.0	39.0
	1995–1999	75.1	53.7	43.2
Belgium^47^	1997–1998	–	–	46.6
Spain^12^	2000–2007	–	–	46.9
Estonia^11^	2005–2009	73.0	–	50.0
Uganda				
Kampala^18^	1993–1997	56.0	13.7	9.1
**Colorectal**				
China				
Guizhou^41^	2013–2015	71.1	49.0	
Cixian, Hebei^42^	2000–2002	39.2	19.6	17.5
Qidong, Jiangsu^39,43^	1993–1997	–	–	25.3
	1998–2002	–	–	29.2
	2001–2007	58.1	42.7	37.7
	2003–2007	–	–	32.2
Japan^35^	1993–1996	83.8	67.5	59.6
Thailand				
Khon Kaen^44^	2003–2012	66.4	44.3	36.9
Europe^45,46^	1990–1994	69.0	49.0	40.0
	1995–1999	71.5	52.1	42.9
Uganda				
Kampala^49^	1993–1997	–	–	6.6
Libya				
Benghazi^48^	2003–2005	–	–	29.5

–No report or non-available in the original articles. OSR, observed survival rate.

**Figure 2:**
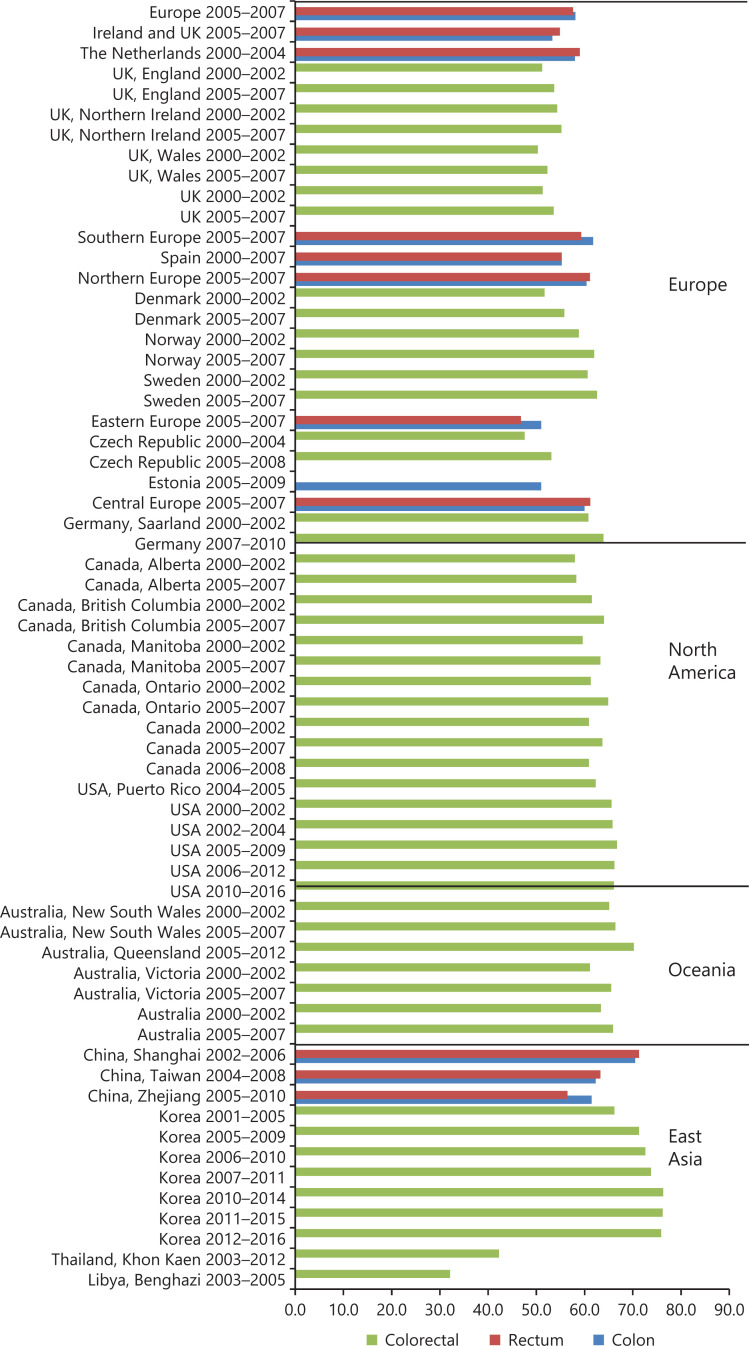
Overall 5-year relative survival rates of colorectal cancer in selected regions during 2000–2016.

**Figure 3:**
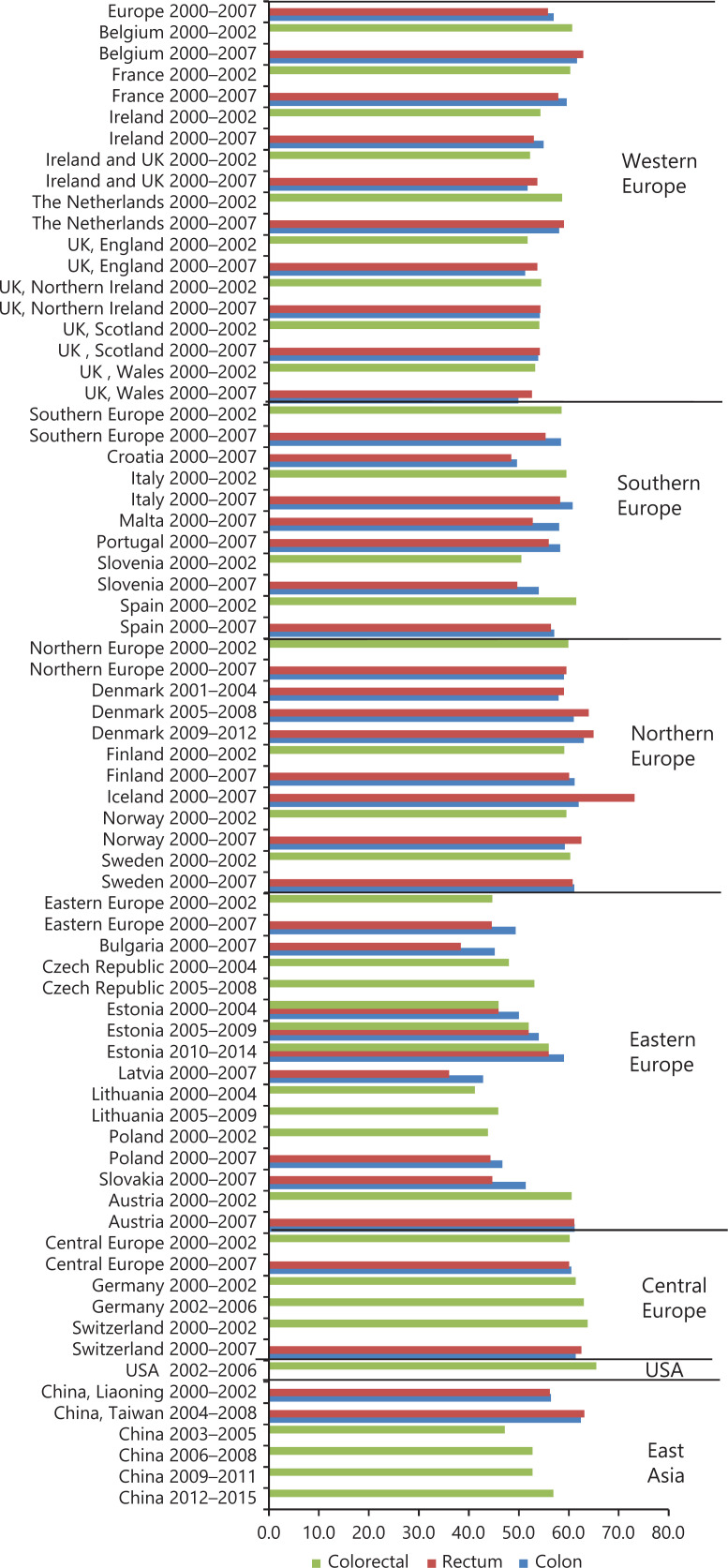
Age-standardized 5-year relative survival rates of colorectal cancer in selected regions during 2000–2016.

**Figures 2 and 3** display the overall and age-standardized 5-year RSRs for CRC in Europe^11,12,14,19,22,28,31,32,34,50–53^, North America^10,19,22,34,54–56^, Oceania^19,57^, Asia^7,36,40,44,58–64^, and Africa^48^. The temporal trend of CRC survival, despite being detected during our analysis, was not displayed due to the time period limitation. For example, the 5-year RSRs in the USA have increased greatly during 1975–2001, from 49.8% to 65.1%, while it barely improved during 2000–2016 (as shown in **Figure 2**). Similarly, the 5-year RSRs in the Republic of Korea have continued to grow during 1993–2010 (see **Supplementary Table S2**), but has remained stable after 2010. In addition, the 5-year RSRs varied widely between regions worldwide. When comparing **Figures 2 and 3**, a slight drop of 5-year RSR was detected after age standardization (for specific data and other details see **Supplementary Tables S3 and S4**). In **Figure 2**, the highest RSRs were observed in the Republic of Korea, Australia, and the USA, while in Thailand and Libya, the RSRs were the lowest. Within Europe (**Figure 3**), the RSRs in eastern countries were relatively poor and varied more noticeably with respect to other European regions.

Regarding colorectal cancer survival by sex, **Table 2** includes the most recent reports of sex-specific 5-year RSRs in selected regions including Japan^30,36^, the Republic of Korea^61^, China^64^, Singapore^18^, Canada^10,21^, USA^21,54^, Brazil^21^, Australia^21^, and Europe^28,46,51^. For colon cancer, the 5-year RSRs were the same in males and females in the USA (1990–1994)^21^, but higher in females from Europe (2005–2007)^28^ and Canada (1990–1994)^21^, and higher in males from Japan (1997–1999)^30^, Singapore (1993–1997)^18^, Brazil (1990–1994)^21^, and Australia (1990–1994)^21^. For rectum cancer, the situation was the opposite. It was unlike that in Japan^30^ and Brazil^21^, but still had the highest rates in men, and the 5-year RSRs in other regions were higher in females than males. For CRC, with the exception of Japan (1997–1999)^36^, the Republic of Korea (2011–2015)^61^, Brazil (1990–1994)^21^, and Eastern Europe (2000–2002)^51^, the 5-year RSRs were higher in females than in males in many countries and regions. It is worth mentioning that the 5-year RSR in the Republic of Korea was 77.9% for males and 73.6% for females during 2011–2015^61^, which was higher than the USA^54^ and China^64^ during similar periods.

**Table 2:** Population-based overall and sex-specific 5-year relative survival rates of colon, rectum, and colorectal cancer in selected regions and periods

Tumor sites & regions	Period	5-year RSRs				
		Age-standardized	Overall	Sex-specific	
				Male	Female
**Colon**					
Japan^30^	1997–1999	68.7	68.9	71.0	66.4
Singapore^18^	1993–1997	49.5	50.2	51.3	49.2
Canada^21^	1990–1994	–	–	56.1*	58.7*
USA^21^	1990–1994	–	–	60.1*	60.1*
Brazil^21^	1990–1994	–	–	33.1*	32.7*
Australia^21^	1990–1994	–	–	57.8*	57.7*
Europe^28^	2005–2007	–	58.1	57.8	58.4
**Rectum**					
Japan^30^	1997–1999	64.7	65.2	65.7	64.5
Singapore^18^	1993–1997	49.0	50.4	48.2	53.4
Canada^21^	1990–1994	–	–	53.1*	58.7*
USA^21^	1990–1994	–	–	56.9*	59.8*
Brazil^21^	1990–1994	–	–	49.3*	38.4*
Australia^21^	1990–1994	–	–	54.8*	59.2*
Europe^28^	2005–2007	–	57.6	57.1	58.7
**Colorectal cancer**					
China^64^	2012–2015	56.9	–	56.3*	57.7
Japan^36^	1997–1999	–	–	68.4*	65.5*
Korea^61^	2011–2015	–	76.2	77.9	73.6
Canada^10^	2006–2008	–	60.9	63.5	64.7
USA^54^	2010–2016	–	66.1	66.0	66.3
Brazil^21^	1990–1994	–	–	47.3*	43.5*
Australia^21^	1990–1994	–	–	56.7*	58.2*
Europe^46^	1995–1999	54.0	53.9	53.4	54.5
Southern Europe^51^	2000–2002	58.6	–	58.2*	59.3*
Northern Europe^51^	2000–2002	59.9	–	58.5*	61.4*
Eastern Europe^51^	2000–2002	44.7	–	46.4*	43.3*
Central Europe^51^	2000–2002	60.2	–	59.1*	61.8*
Ireland and UK^51^	2000–2002	52.3	–	51.3*	53.7*

–No report or non-available in the original articles. *Age-standardized RSR. RSR, relative survival rate.

### Colorectal cancer survival by age

The age-specific RSRs of CRC are included in **Supplementary Tables S5 and S6**, which showed age-specific 5-year RSRs of colon, rectum, and colorectal cancers in Asia^18,35,37,39–40,43^, North America^10,18,27,34,54^, Europe^13,23,28,34,45–46^, and Africa^18^. According to the outcomes, besides the oldest age group (≥ 75 years of age) displaying the poorest 5-year RSR, there was a relatively weak decreasing trend of RSR in other age groups (≤ 44, 45–54, 55–64, and 65–74 years of age). In most countries and regions of Asia and North America, the highest RSRs occurred in the following age groups: colon cancer, < 44 and 45–54 years of age; rectum cancer, 45–54 and 55–64 years of age; and CRC, 55–64 years of age. Nevertheless, whether colon, rectum, or colorectal cancer, the 5-year RSRs invariably decreased with age in Europe, with the highest survival rate at ≤ 44 years of age.

## Discussion

The aim of our systematic review was to collect and evaluate the survival data of colorectal cancer patients from population-based cancer registries, other than clinical trials or hospital-based survival studies. The statistics from cancer registries should be used as prognostic measurements for cancer in patients at the population level. Estimates regarding survival, such as “observed survival rate, relative survival rate, and age-standardized relative survival rate” were selected in our study. Global patterns and trends of CRC survival were also included in our report. Additionally, we further compared the data of observed and relative survival rates of CRC by periods, regions, sexes, age groups, and tumor sites.

CRC is currently considered as one of the most distinct of the cancers, replacing infection-related cancers in countries experiencing rapid social and economic changes together with other cancers predominantly related to western lifestyles, which are frequently found in high income countries. The chronological tendencies of CRC patterns are mainly divided into 3 types^2^: (1) increasing incidence accompanied by increasing mortality, represented by some medium and high HDI countries including Brazil, China, Colombia, and Poland; (2) increasing incidence accompanied by decreasing mortality, as seen in some high HDI countries such as UK, Canada, Denmark, and Singapore; and (3) decreases in both incidence and mortality, represented by the highest HDI countries, such as Australia, France, Japan, and the USA. Therefore, it is of great importance to compare and evaluate evidence of survival for colorectal cancer worldwide.

Unlike patterns of incidence and mortality, the survival of CRC has increased with time, generally as a result of the improved sensitivities of diagnostic and staging procedures, refined surgical techniques, use of preoperative radiation for rectum cancer, the introduction of novel agents, and regimens for chemotherapy^28^. Since past decades, especially in patients with locally or regionally spread cancers, survival rates have dramatically improved. Nevertheless, limited progress has been observed in patients with advanced and metastatic disease^65,66^ (approximately 45% of patients diagnosed with CRC die due to the disease, despite treatment^67^). According to the available published data (starting from the 1960s), the survival rates of CRC are showing a remarkable upward trend from the 1960s to 1990s. However, this trend began to gradually stabilize after the 21st century. In terms of regional disparities in survival, our study showed relatively higher survival rates in the United States, Canada, Australia, Japan, and the Republic of Korea. A certain variability was also registered within Europe; Estonia, Lithuania, and Poland in Eastern Europe had significantly poorer survival rates than other European countries and regions. These differences may be attributable to the socio-economic status and related medical technology and investment in healthcare^68^. Additionally, in most countries and regions, the survival rates of colon cancer are slightly higher than those of rectum cancer. However, in many developed countries, colon and rectum cancer showed similar patterns of 5-year relative survival or even higher estimates for rectum cancer^69^.

The existence of CRC screening programs, to detect tumors at earlier stages, is considered of central importance in assessing prognosis for cancer worldwide^70^. Since the late 1980s, several developed countries have begun to conduct screening programs for colorectal cancer. Although the range of CRC screening modalities has currently expanded, and many population-based programs have been implemented over the past decades, large geographical areas are still not benefiting from the implementation of CRC screening^65,70^. For example, in Poland, despite an increase in funding, the percentage of the population participating in the program was very low and accounted for only 16.8% of the target group^71^. As expected, in Western countries, where CRC incidence is higher, the resources allocated to the screening program allowed a better implementation, with an early detection and final benefits in terms of survival and mortality rates.

The disparities in sex and age in CRC survival rates were also included in our study. In terms of sex-specific RSRs, we observed that the 5-year RSRs of rectum cancer and colorectal cancer were higher in females than in males in most countries and regions, whereas the pattern in colon cancer seemed to be the opposite. However, a study from the EUROCARE suggested that women had a survival advantage for most cancers (17/26 sites)^72^, including colon and rectum cancers. Although the age group with the highest survival rate varied between different regions, cancer patients > 75 years of age always had the poorest survival rate. This may be due to the presence of comorbidities and various chronic diseases, as observed in survival studies. Furthermore, available treatment guidelines might be less effective for elderly patients, as these patients are often underrepresented in clinical trials^28^.

## Conclusions

In conclusion, we summarized 1–5-year OSRs and RSRs of CRC, which showed variations in geographic, temporal, sex, age gradient, and tumor sites. Except for clinical and treatment prognostic factors, our study implied that the region, period, sex, and age were also associated with the population-based survival rate of CRC. Therefore, CRC prevention and screening in etiological, basic science, and clinical research should be more comprehensive and frequent.
