# Supplementary material for: Global pattern and trends of colorectal cancer survival: a systematic review of population-based registration data
Source: Cancer Biol Med. 2021 Sep 6;19(2):175–86. doi: 10.20892/j.issn.2095-3941.2020.0634 (PMC8832952; doi:10.20892/j.issn.2095-3941.2020.0634)
Supplement: Supplementary file 1 [file cbm-19-175-s001.pdf]

Supplementary materials

Table S1 All countries and regions involved

Continent	Country	Area
Asia	China	Shanghai
		Zhejiang
		Tianjin
		Liaoning
		Guizhou
		Cixian, Hebei
		Qidong, Jiangsu
		Hong Kong
		Taiwan
	Japan	
	Korea	Seoul
		Busan
		Incheon
		Jeju Island
	Philippine	Metro Manila &Rizal Province
	Singapore	
	Thailand	Songkhla
		Lampang
		Chiang Mai
		Khon Kaen
	India	Bhopal
		Barshi
		Mumbai
		Karunagappally
	Turkey	Izmir
North America	USA	Oklahoma
		Puerto Rico
	Canada	Alberta
		British Columbia
		Manitoba
		Ontario
	Cuba	
South America	Brazil	
Oceania	Australia	New South Wales
		Queensland
		Victoria
Europe	Southern Europe	
	Northern Europe	
	Eastern Europe	
	Central Europe	
	Ireland and UK	
	Belgium	
	France	
	Ireland	
	The Netherlands	Eindhoven
		North-Holland and Flevoland
	UK	England
		Scotland
		Wales
		Northern Ireland
	Croatia	
	Italy	Torino
		Tuscany
	Malta	
	Portugal	
	Slovenia	
	Spain	
	Denmark	
	Finland	
	Iceland	
	Norway	
	Sweden	
	Bulgaria	
	Czech Republic	
	Estonia	
	Latvia	

Table S1 Continued

Continent	Country	Area
	Lithuania	
	Poland	Cracow
	Slovakia	
	Germany	Munich
		Saarland
	Switzerland	Geneva
	Austria	
Africa	Uganda	Kampala
	Libya	Benghazi

Table S2 Population-based overall 1-, 3-, and 5-year relative survival rates and age-standardized 1- and 5-year relative survival rates of colorectal cancer

Regions	Period	RSR (%)			Age-standardized RSR (%)	
		1-year	3-year	5-year	1-year	5-year
Asia						
China[64]	2003–2005	–	–	–	–	47.2
	2006–2008	–	–	–	–	52.7
	2009–2011	–	–	–	–	52.7
	2012–2015	–	–	–	–	56.9
Guizhou[41]	2013–2015	84.0	77.4	–	–	–
Cixian, Hebei[42]	2000–2002	40.2	21.3	20.2	–	–
Qidong, Jiangsu[39,43]	1993–1997	–	–	32.3	–	–
	1998–2002	–	–	37.0	–	–
	2001–2007	61.0	49.6	48.7	–	–
	2003–2007	–	–	42.7	–	–
Japan[35]	1993–1996	86.0	73.0	68.1	–	–
Korea[37,58-62]	1993–1995	–	–	54.8	–	–
	1996–2000	–	–	58.0	–	–
	2001–2005	–	–	66.6	–	–
	2005–2009	–	–	71.3	–	–
	2006–2010	–	–	72.6	–	–
	2007–2011	–	–	73.8	–	–
	2010–2014	–	–	76.3	–	–
	2011–2015	–	–	76.2	–	–
	2012–2016	–	–	75.9	–	–

Table S2 Continued

Regions	Period	RSR (%)			Age-standardized RSR (%)	
		1-year	3-year	5-year	1-year	5-year
Jeju Island[38]	2000–2001	81.4	62.6	–	–	–
Philippines[17,25]	1995–1999	–	–	37.8	–	–
	1998–2002	–	–	37.0	–	–
Metro Manila & Rizal Province[74]	1998–2002	–	–	–	–	40.2
Thailand						
Khon Kaen[44]	2003–2012	68.4	47.9	42.3	–	–
North America						
USA[22,31,34,54,55,75-77]	1990–1992	–	–	61.2	–	–
	1990–1999	–	–	62.0	–	–
	1993–1995	–	–	59.9	–	–
	1995–2000	–	–	63.4	–	–
	1996–1998	–	–	62.4	–	–
	1999–2001	–	–	65.1	–	–
	2000–2002	–	–	65.6	–	–
	2001–2004	–	–	50.6	–	–
	2002–2004	–	–	65.8	–	–
	2002–2006	–	–	–	–	65.5
	2005–2008	–	–	52.8	–	–
	2005–2009	–	–	66.7	–	–
	2006–2012	–	–	66.2	–	–
	2007–2010	–	–	66.0	–	–
	2010–2016	–	–	66.1	–	–
Oklahoma[77]	1997–2000	–	–	46.5	–	–
Puerto Rico[56]	2004–2005	81.9	68.4	62.3	–	–
Canada[10,15,19]	1992–1994	–	–	–	–	56.0
	1995–1999	79.1	–	58.1	–	–
	2000–2002	81.5	–	60.9	–	–
	2005–2007	83.5	–	63.7	–	–
	2006–2008	82.2	–	60.9	–	–
Alberta[19]	1995–1999	77.6	–	56.3	–	–
	2000–2002	79.8	–	58.0	–	–
	2005–2007	80.4	–	58.3	–	–
British Columbia[19]	1995–1999	80.7	–	59.8	–	–
	2000–2002	82.4	–	61.5	–	–

Table S2 Continued

Regions	Period	RSR (%)			Age-standardized RSR (%)	
		1-year	3-year	5-year	1-year	5-year
	2005–2007	84.3	–	64.0	–	–
Manitoba[19]	1995–1999	79.8	–	57.6	–	–
	2000–2002	80.4	–	59.6	–	–
	2005–2007	82.1	–	63.3	–	–
Ontario[19]	1995–1999	78.7	–	58.0	–	–
	2000–2002	81.7	–	61.3	–	–
	2005–2007	83.9	–	64.9	–	–
Oceania						
Australia[19]	1995–1999	80.0	–	60.0	–	–
	2000–2002	82.5	–	63.4	–	–
	2005–2007	84.9	–	65.9	–	–
New South Wales[19]	1995–1999	80.3	–	61.2	–	–
	2000–2002	83.2	–	65.1	–	–
	2005–2007	84.7	–	66.4	–	–
Queensland[57]	1997–2004	–	–	64.6	–	–
	2005–2012	–	–	70.2	–	–
Victoria[19]	1995–1999	79.6	–	58.4	–	–
	2000–2002	81.6	–	61.1	–	–
	2005–2007	85.1	–	65.5	–	–
Europe						
Europe[45,46]	1990–1994	72.0	56.0	50.0	–	–
	1995–1999	74.7	59.6	53.9	75.8	54.0
Southern Europe[51]	2000–2002	–	–	–	–	58.6
Northern Europe[51]	2000–2002	–	–	–	–	59.9
Eastern Europe[51]	2000–2002	–	–	–	–	44.7
Central Europe[51]	2000–2002	–	–	–	–	60.2
Ireland and UK[51]	2000–2002	–	–	–	–	52.3
Belgium[46,51]	1995–1999	–	–	–	79.4	57.4
	2000–2002	–	–	–	–	60.7
France[46,51]	1995–1999	–	–	–	80.6	57.9
	2000–2002	–	–	–	–	60.3
Ireland[46,51]	1995–1999	–	–	–	72.3	50.6
	2000–2002	–	–	–	–	54.3
The Netherlands[46,51]	1995–1999	–	–	–	77.5	57.1
	2000–2002	–	–	–	–	58.7

Table S2 Continued

Regions	Period	RSR (%)			Age-standardized RSR (%)	
		1-year	3-year	5-year	1-year	5-year
Eindhoven[78]	2000	–	–	–	–	56.2
	2004	–	–	–	–	56.1
North-Holland and Flevoland[75]	1999–2001	–	64.0	–	–	–
UK[19]	1995–1999	70.2	–	47.8	–	–
	2000–2002	73.0	–	51.3	–	–
	2005–2007	74.7	–	53.6	–	–
South East England[79]	2002–2006	68.8	–	–	71.1	–
England[19,46,51]	1995–1999	70.2	–	47.8	72.2	50.5
	2000–2002	72.9	–	51.2	–	51.8
	2005–2007	74.7	–	53.7	–	–
Scotland[46,51,78]	1995–1999	–	–	–	73.3	51.5
	2000	–	–	–	–	52.0
	2000–2002	–	–	–	–	54.1
	2004	–	–	–	–	55.0
Wales[19,46,51]	1995–1999	68.3	–	45.9	74.2	50.6
	2000–2002	72.3	–	50.3	–	53.3
	2005–2007	73.6	–	52.3	–	–
Northern Ireland[19,46,51]	1995–1999	74.3	–	51.5	74.5	51.8
	2000–2002	76.8	–	54.3	–	54.5
	2005–2007	76.2	–	55.2	–	–
Italy[46,51]	1995–1999	–	–	–	78.5	57.1
	2000–2002	–	–	–	–	59.5
Torino[78]	2000	–	–	–	–	58.9
	2004	–	–	–	–	57.7
Tuscany[78]	2000	–	–	–	–	56.4
	2004	–	–	–	–	62.6
Malta[46]	1995–1999	–	–	–	73.8	51.2
Portugal[46]	1995–1999	–	–	–	75.1	51.0
Slovenia[46,51,78]	2000	–	–	–	–	44.1
	2004	–	–	–	–	48.2
	1995–1999	–	–	–	68.4	44.2
	2000–2002	–	–	–	–	50.5
Spain[46,51]	1995–1999	–	–	–	75.9	53.6
	2000–2002	–	–	–	–	61.5

Table S2 Continued

Regions	Period	RSR (%)			Age-standardized RSR (%)	
		1-year	3-year	5-year	1-year	5-year
Denmark[19,46,51]	1995–1997	–	–	46.8	–	–
	1995–1999	71.7	–	48.2	73.1	49.3
	2000–2002	73.9	–	51.7	–	–
	2005–2007	77.7	–	55.8	–	–
Finland[46,51,78]	2000	–	–	–	–	55.8
	2004	–	–	–	–	59.9
	1995–1999	–	–	–	79.0	57.7
	2000–2002	–	–	–	–	59.1
Iceland[46]	1995–1999	–	–	–	78.9	57.1
Norway[19,46,51,78]	2000	–	–	–	–	57.5
	2004	–	–	–	–	60.9
	1995–1999	78.6	–	56.9	79.0	58.3
	2000–2002	78.7	–	58.8	–	59.5
	2005–2007	82.4	–	62.0	–	–
Sweden[19,46,51]	1995–1999	81.8	–	58.5	81.2	58.5
	2000–2002	82.8	–	60.6	–	60.3
	2005–2007	83.8	–	62.6	–	–
Czech Republic[14,46,51]	1995–1999	–	–	–	67.8	43.9
	2000–2002	–	–	–	–	46.3
	2000–2004	–	–	47.6	–	48.0
	2005–2008	–	–	53.1	–	53.1
Estonia[32,78]	2000	–	–	–	–	47.2
	2004	–	–	–	–	49.4
	1995–1999	–	–	–	–	38.0
	2000–2004	–	–	–	–	46.0
	2005–2009	–	–	–	–	52.0
	2010–2014	–	–	–	–	56.0
Lithuania[52,78]	2000	–	–	–	–	40.1
	2004	–	–	–	–	44.8
	1995–1999	–	–	–	–	34.0
	2000–2004	–	–	–	–	41.2
	2005–2009	–	–	–	–	45.9
Poland[46,51]	1995–1999	–	–	–	64.0	38.8
	2000–2002	–	–	–	–	43.8

Table S2 Continued

Regions	Period	RSR (%)			Age-standardized RSR (%)	
		1-year	3-year	5-year	1-year	5-year
Cracow[78]	2000	–	–	–	–	35.4
	2004	–	–	–	–	41.9
Germany[13,31,46,51]	1995–1999	–	–	–	78.6	57.5
	2000–2002	–	–	–	–	61.4
	2002–2006	–	–	–	–	63.0
	2007–2010	–	–	63.9	–	–
Saarland[22,78]	2000	–	–	–	–	59.1
	2004	–	–	–	–	64.7
	2000–2002	–	–	60.8	–	–
Switzerland[46,51]	1995–1999	–	–	–	81.6	59.9
	2000–2002	–	–	–	–	63.8
Geneva[78]	2000	–	–	–	–	60.7
	2004	–	–	–	–	66.2
Austria[46,51]	1995–1999	–	–	–	77.3	58.1
	2000–2002	–	–	–	–	60.6
Africa						
Uganda						
Kampala[49]	1993–1997	–	–	8.3	–	–
Libya						
Benghazi[48]	2003–2005	–	–	32.1	–	–

–No report or non-available in the original articles.

Table S3 Population-based overall 1-, 3-, and 5-year relative survival rates and age-standardized 1- and 5-year relative survival rates of colon cancer

Regions	Period	RSR (%)			Age-standardized RSR (%)	
		1-year	3-year	5-year	1-year	5-year
Asia						
China						
Shanghai[20]	1992–1995	–	–	50.6	–	–
Zhejiang[40]	2005–2010	78.1	65.1	61.5	–	–
Tianjin[20]	1991–1999	71.6	60.9	61.4	–	61.3
Liaoning[36]	2000–2002	–	–	–	–	56.4
Hong Kong[18]	1996–2001	78.2	65.2	61.3	–	61.0
Taiwan[63]	2004–2008	–	–	62.3	–	62.4

Table S3 Continued

Regions	Period	RSR (%)			Age-standardized RSR (%)	
		1-year	3-year	5-year	1-year	5-year
Japan[30,35]	1993–1996	–	–	69.8	–	–
	1997–1999	–	–	68.9	–	68.7
Korea						
Seoul[18]	1993–1997	78.9	66.7	64.1	–	59.8
Busan[18]	1996–2001	76.4	60.6	54.4	–	46.6
Incheon[18]	1997–2001	71.4	58.6	54.5	–	49.5
Philippine[25]	1998–2002	–	–	37.3	–	–
Singapore[18]	1993–1997	73.0	56.9	50.2	–	49.5
Thailand						
Songkhla[18]	1990–1999	78.9	53.6	48.3	–	45.4
Lampang[18]	1990–2000	58.1	43.5	38.0	–	35.0
Chiang Mai[18]	1993–1997	62.1	40.8	30.8	–	27.8
Khon Kaen[18]	1993–1997	65.1	51.8	42.8	–	38.7
India						
Bhopal[18]	1991–1995	53.9	27.1	7.0	–	3.2
Mumbai[18]	1992–1999	54.5	38.8	32.3	–	25.4
Turkey						
Izmir[18]	1995–1997	76.0	58.7	52.5	–	53.2
North America						
Canada[27]	1992–1994	–	–	56.0	–	–
	1995–1997	–	–	57.3	–	–
	1998–2000	–	–	58.2	–	58.4
Cuba[18]	1994–1995	60.6	43.3	41.2	–	40.5
Europe						
Europe[28,45,46]	1990–1994	70.0	56.0	51.0	–	–
	1995–1999	72.7	58.9	54.3	74.2	54.5
	1999–2001	–	–	54.2	–	–
	2000–2007	–	–	–	77.6	57.0
	2005–2007	–	–	58.1	–	–
Southern Europe[28]	1999–2001	–	–	57.5	–	–
	2000–2007	–	–	–	78.6	58.5
	2005–2007	–	–	61.8	–	–
Northern Europe[28]	1999–2001	–	–	55.9	–	–
	2000–2007	–	–	–	79.3	59.0
	2005–2007	–	–	60.4	–	–

Table S3 Continued

Regions	Period	RSR (%)			Age-standardized RSR (%)	
		1-year	3-year	5-year	1-year	5-year
Eastern Europe[28]	1999–2001	–	–	46.2	–	–
	2000–2007	–	–	–	69.7	49.4
	2005–2007	–	–	51.0	–	–
Central Europe[28]	1999–2001	–	–	56.8	–	–
	2000–2007	–	–	–	80.5	60.5
	2005–2007	–	–	60.0	–	–
Ireland and UK[28]	1999–2001	–	–	49.7	–	–
	2000–2007	–	–	–	72.6	51.8
	2005–2007	–	–	53.3	–	–
Belgium[28,46]	1995–1999	–	–	–	76.5	56.4
	2000–2007	–	–	–	82.9	61.7
France[28,46]	1995–1999	–	–	–	78.8	58.0
	2000–2007	–	–	–	81.6	59.6
Ireland[28,46]	1995–1999	–	–	–	71.6	52.2
	2000–2007	–	–	–	75.1	55.0
The Netherlands[28,46,50]	1995–1999	–	–	58.0	76.0	56.8
	2000–2004	–	–	58.0	–	–
	2000–2007	–	–	–	78.0	58.1
UK						
England[28,46]	1995–1999	–	–	–	69.9	49.9
	2000–2007	–	–	–	72.2	51.3
Scotland[28,46]	1995–1999	–	–	–	72.1	51.6
	2000–2007	–	–	–	74.6	53.9
Wales[28,46]	1995–1999	–	–	–	72.2	50.8
	2000–2007	–	–	–	70.4	49.9
Northern Ireland[28,46]	1995–1999	–	–	–	73.2	53.4
	2000–2007	–	–	–	74.9	54.2
Croatia[28]	2000–2007	–	–	–	66.1	49.6
Italy[28,46]	1995–1999	–	–	–	78.1	58.7
	2000–2007	–	–	–	81.3	60.8
Malta[28,46]	1995–1999	–	–	–	71.5	49.7
	2000–2007	–	–	–	74.9	58.1
Portugal[28,46]	1995–1999	–	–	–	74.2	51.9
	2000–2007	–	–	–	78.6	58.3

Table S3 Continued

Regions	Period	RSR (%)			Age-standardized RSR (%)	
		1-year	3-year	5-year	1-year	5-year
Slovenia[28,46]	1995–1999	–	–	–	66.1	45.8
	2000–2007	–	–	–	73.9	54.0
Spain[12,23,28,46]	1995–1999	–	–	54.7	74.7	54.9
	2000–2007	–	–	55.3	77.4	57.1
Denmark[28,29,46,53]	1995–1997	–	–	46.9	–	–
	1995–1999	–	–	–	70.9	49.3
	2000–2007	–	–	–	73.7	53.6
	2001–2004	–	–	–	76.0	58.0
	2005–2008	–	–	–	78.0	61.0
	2009–2012	–	–	–	80.0	63.0
Finland[16,26,28,46]	1997	–	–	59.4	–	–
	1990–1992	–	–	52.7	–	–
	1995–1999	–	–	–	77.5	59.1
	2000–2007	–	–	–	80.4	61.2
Iceland[28,46]	1995–1999	–	–	–	78.7	56.9
	2000–2007	–	–	–	81.2	62.0
Norway[28,46]	1995–1999	–	–	–	77.1	57.0
	2000–2007	–	–	–	79.2	59.2
Sweden[24,28,46]	1990–1994	74.5	60.2	54.5	–	–
	1995–1999	78.0	63.2	57.2	79.7	57.7
	2000–2007	–	–	–	82.5	61.1
Bulgaria[28]	2000–2007	–	–	–	65.8	45.2
Czech Republic[28,46]	1995–1999	–	–	–	66.5	46.9
	2000–2007	–	–	–	72.5	52.5
Estonia[11,28,32,33]	1997	63.0	–	51.0	63.0	51.0
	1995–1999	–	–	–	–	50.0
	2000–2004	–	–	–	–	50.0
	2000–2007	–	–	–	71.3	51.7
	2005–2009	71.0	–	51.0	–	54.0
	2010–2014	–	–	–	–	59.0
Latvia[28]	2000–2007	–	–	–	64.3	42.9
Lithuania[28]	2000–2007	–	–	–	66.0	47.1
Poland[28,46]	1995–1999	–	–	–	60.1	38.7
	2000–2007	–	–	–	68.8	46.7

Table S3 Continued

Regions	Period	RSR (%)			Age-standardized RSR (%)	
		1-year	3-year	5-year	1-year	5-year
Slovakia[28]	2000–2007	–	–	–	72.0	51.4
Germany[13,28,46]	1995–1999	–	–	–	76.9	57.9
	2000–2007	–	–	–	81.5	62.2
	2002–2006	–	–	–	–	63.8
Switzerland[28,46]	1995–1999	–	–	–	79.7	59.1
	2000–2007	–	–	–	82.9	61.4
Austria[28,46]	1995–1999	–	–	–	75.7	58.7
	2000–2007	–	–	–	79.5	61.2
Africa						
Uganda						
Kampala[18]	1993–1997	57.2	22.7	7.4	–	6.2

–No report or non-available in the original articles.

Table S4 Population-based overall 1-, 3-, and 5-year relative survival rates and age-standardized 1- and 5-year relative survival rates of rectum cancer

Regions	Period	RSR (%)			Age-standardized RS (%)	
		1-year	3-year	5-year	1-year	5-year
Asia						
China						
Shanghai[20]	1992–1995	–	–	47.7	–	–
Zhejiang[40]	2005–2010	79.6	62.2	56.5	–	–
Tianjin[20]	1991–1999	75.4	60.5	56.0	–	53.0
Liaoning[36]	2000–2002	–	–	–	–	56.2
Hong Kong[18,20]	1996–2001	82.4	66.4	60.6	–	59.2
Taiwan[63]	2004–2008	–	–	63.3	–	63.1
Japan[30,35]	1993–1996	–	–	65.2	–	–
	1997–1999	–	–	65.2	–	64.7
Korea						
Seoul[18]	1993–1997	84.2	65.8	60.6	–	57.5
Busan[18]	1996–2001	84.1	63.3	54.7	–	44.5
Incheon[18]	1997–2001	79.4	62.6	55.4	–	46.7
Philippine[25]	1998–2002	–	–	37.9	–	–
Singapore[18]	1993–1997	76.1	58.0	50.4	–	49.0
Thailand						

Table S4 Continued

Regions	Period	RSR (%)			Age-standardized RS (%)	
		1-year	3-year	5-year	1-year	5-year
Songkhla[18]	1990–1999	74.9	46.4	35.4	–	36.2
Lampang[18]	1990–2000	66.7	44.7	38.9	–	42.3
Chiang Mai[18]	1993–1997	70.5	38.3	29.7	–	28.7
Khon Kaen[18]	1993–1997	71.1	45.7	42.5	–	42.5
India						
Bhopal[18]	1991–1995	58.0	27.2	8.5	–	4.0
Barshi[18]	1993–2000	47.8	18.4	13.0	–	9.2
Mumbai[18]	1992–1999	61.9	41.2	33.6	–	26.1
Karunagappally[18]	1991–1997	79.2	55.7	33.4	–	43.6
Turkey						
Izmir[18]	1995–1997	85.0	60.1	50.4	–	51.6
North America						
Canada[27]	1992–1994	–	–	54.5	–	–
	1995–1997	–	–	57.1	–	–
	1998–2000	–	–	59.7	–	59.8
Cuba[18]	1994–1995	72.8	50.9	48.5	–	47.6
Europe						
Europe[28,45,46]	1990–1994	75.0	56.0	48.0	–	–
	1995–1999	78.2	60.8	53.6	78.7	53.2
	1999–2001	–	–	52.1	–	–
	2000–2007	–	–	–	80.8	55.8
	2005–2007	–	–	57.6	–	–
Southern Europe[28]	1999–2001	–	–	53.5	–	–
	2000–2007	–	–	–	80.4	55.4
	2005–2007	–	–	59.3	–	–
Northern Europe[28]	1999–2001	–	–	55.6	–	–
	2000–2007	–	–	–	83.4	59.5
	2005–2007	–	–	61.1	–	–
Eastern Europe[28]	1999–2001	–	–	39.7	–	–
	2005–2007	–	–	46.8	–	–
	2000–2007	–	–	–	72.4	44.6
Central Europe[28]	1999–2001	–	–	55.9	–	–
	2000–2007	–	–	–	83.7	60.1
	2005–2007	–	–	61.2	–	–

Table S4 Continued

Regions	Period	RSR (%)			Age-standardized RS (%)	
		1-year	3-year	5-year	1-year	5-year
Ireland and UK[28]	1999–2001	–	–	50.9	–	–
	2000–2007	–	–	–	78.5	53.7
	2005–2007	–	–	54.9	–	–
Belgium[28,46,47]	1995–1999	–	–	–	83.7	58.5
	1997–1998	–	–	–	–	58.5
	2000–2007	–	–	–	86.6	62.9
France[28,46]	1995–1999	–	–	–	83.3	57.6
	2000–2007	–	–	–	83.7	57.9
Ireland[28,46]	1995–1999	–	–	–	73.9	48.5
	2000–2007	–	–	–	78.4	53.0
The Netherlands[28,46,50]	1995–1999	–	–	57.0	79.9	57.2
	2000–2004	–	–	59.0	–	–
	2000–2007	–	–	–	82.7	59.0
	2005–2006	–	–	–	–	–
UK						
England[28,46]	1995–1999	–	–	–	76.3	51.8
	2000–2007	–	–	–	78.3	53.7
Scotland[28,46]	1995–1999	–	–	–	76.3	51.5
	2000–2007	–	–	–	79.4	54.2
Wales[28,46]	1995–1999	–	–	–	77.4	50.7
	2000–2007	–	–	–	78.6	52.6
Northern Ireland[28,46]	1995–1999	–	–	–	78.3	49.5
	2000–2007	–	–	–	80.1	54.3
Croatia[28]	2000–2007	–	–	–	71.4	48.5
Italy[28,46]	1995–1999	–	–	–	80.3	54.3
	2000–2007	–	–	–	83.3	58.3
Malta[28,46]	1995–1999	–	–	–	77.3	53.5
	2000–2007	–	–	–	82.1	52.8
Portugal[28,46]	1995–1999	–	–	–	76.6	49.6
	2000–2007	–	–	–	81.4	56.0
Slovenia[28,46]	1995–1999	–	–	–	71.2	42.6
	2000–2007	–	–	–	76.5	49.7
Spain[12,23,28,46]	1995–1999	–	–	50.2	78.1	51.7
	2000–2007	–	–	55.3	81.2	56.4

Table S4 Continued

Regions	Period	RSR (%)			Age-standardized RS (%)	
		1-year	3-year	5-year	1-year	5-year
Denmark[28,29,46,53]	1995–1997	–	–	46.6	–	–
	1995–1999	–	–	–	76.0	49.2
	2000–2007	–	–	–	80.3	54.6
	2001–2004	–	–	–	81.0	59.0
	2005–2008	–	–	–	84.0	64.0
	2009–2012	–	–	–	85.0	65.0
Finland[16,26,28,46]	1997	–	–	55.7	–	–
	1990–1992	–	–	48.0	–	–
	1995–1999	–	–	–	81.0	55.2
	2000–2007	–	–	–	82.6	60.1
Iceland[28,46]	1995–1999	–	–	–	76.0	54.4
	2000–2007	–	–	–	86.7	73.2
Norway[28,46]	1995–1999	–	–	–	82.2	60.4
	2000–2007	–	–	–	84.5	62.5
Sweden[24,28,46]	1990–1994	79.4	61.3	53.6	–	–
	1995–1999	81.3	65.4	57.6	83.7	59.7
	2000–2007	–	–	–	85.3	60.8
Bulgaria[28]	2000–2007	–	–	–	67.5	38.4
Czech Republic[28,46]	1995–1999	–	–	–	69.5	39.9
	2000–2007	–	–	–	75.2	48.7
Estonia[11,28,32,33]	1997	66.0	–	38.0	66.0	37.0
	1995–1999	–	–	–	–	38.0
	2000–2004	–	–	–	–	46.0
	2000–2007	–	–	–	73.5	47.9
	2005–2009	–	–	–	–	52.0
	2010–2014	–	–	–	–	56.0
Latvia[28]	2000–2007	–	–	–	69.2	36.1
Lithuania[28]	2000–2007	–	–	–	67.9	43.0
Poland[28,46]	1995–1999	–	–	–	70.4	38.9
	2000–2007	–	–	–	73.2	44.3
Slovakia[28]	2000–2007	–	–	–	73.6	44.7
Germany[13,28,46]	1995–1999	–	–	–	81.2	56.4
	2000–2007	–	–	–	83.4	60.2

Table S4 Continued

Regions	Period	RSR (%)			Age-standardized RS (%)	
		1-year	3-year	5-year	1-year	5-year
Munich[73]	1996–1998	–	–	62.2	–	–
Switzerland[28,46]	1995–1999	–	–	–	84.7	60.7
	2000–2007	–	–	–	87.4	62.5
Austria[28,46]	1995–1999	–	–	–	79.6	56.8
	2000–2007	–	–	–	83.1	61.1
	2002–2006	–	–	–	–	61.2
Africa						
Uganda						
Kampala[18]	1993–1997	58.4	15.4	11.0	–	7.2

–No report or non-available in the original articles.

Table S5 Population-based age-specific 5-year relative survival rates of colon and rectum cancers during 1990–2016

Regions	Period	Colon					Rectum				
		≤ 44	45–54	55–64	65–74	≥ 75	≤ 44	45–54	55–64	65–74	≥ 75
Asia											
China											
Tianjin[18]	1991–1999	62.7	65.6	60.1	61.6	62.9	55.4	57.3	58.8	59.6	45.3
Hong Kong[18]	1996–2001	64.3	63.3	64.0	62.3	58.1	62.5	65.5	64.7	61.8	53.1
Korea											
Seoul[18]	1993–1997	69.5	67.3	67.4	62.2	51.0	59.8	63.0	62.9	60.6	52.0
Busan[18]	1996–2001	60.6	67.4	58.8	48.5	30.9	53.7	59.8	63.4	53.0	23.4
Incheon[18]	1997–2001	68.2	63.8	56.5	50.1	38.1	53.7	58.9	67.3	50.1	28.0
Singapore[18]	1993–1997	55.4	56.0	54.6	47.8	45.6	54.0	52.4	57.0	47.3	44.5
Thailand											
Songkhla[18]	1990–1999	54.9	61.8	44.9	54.1	32.9	32.6	34.4	43.7	20.7	43.8
Lampang[18]	1990–2000	40.6	43.2	36.9	38.9	28.9	38.9	50.2	38.4	27.8	51.1
Chiang Mai[18]	1993–1997	26.2	33.4	46.5	24.0	19.8	29.6	37.7	30.4	28.5	23.3
Khon Kaen[18]	1993–1997	44.7	47.6	38.4	47.4	33.0	28.3	66.9	40.4	39.9	42.9
India											
Bhopal[18]	1991–1995	12.7	15.1	0.0	0.0	0.0	14.6	10.5	7.8	0.0	0.0
Barshi[18]	1993–2000	–	–	–	–	–	0.0	42.8	0.0	21.8	0.0
Mumbai[18]	1992–1999	44.1	42.5	27.9	18.8	17.6	43.2	41.9	32.5	16.2	16.0
Karunagappally[18]	1991–1997	–	–	–	–	–	24.2	31.6	26.9	42.1	–

Table S5 Continued

Regions	Period	Colon					Rectum				
		≤ 44	45–54	55–64	65–74	≥ 75	≤ 44	45–54	55–64	65–74	≥ 75
Turkey											
Izmir[18]	1995–1997	48.0	57.8	51.8	51.4	55.6	39.4	48.3	47.7	64.5	53.0
North America											
USA[54]	2010–2016	69.5	71.2	67.0	66.4	55.2	–	–	–	–	–
Canada[27]	1992–1994	61.1	60.9	61.3	59.2	55.7	64.9	63.4	62.7	61.5	54.0
Cuba[18]	1994–1995	51.8	40.8	43.3	49.7	31.7	52.0	52.9	52.6	47.5	42.9
Europe											
Europe[28,45,46]	1990–1994	59.0	56.0	54.0	51.0	46.0	54.0	55.0	52.0	50.0	39.0
	1995–1999	62.7	57.4	56.8	54.8	49.2	60.5	59.5	57.4	53.7	45.0
	1999–2001	63.7	57.3	57.0	55.0	47.7	60.2	59.3	57.2	53.0	42.1
	2000–2007	64.7	61.0	61.6	57.7	49.3	63.9	62.8	62.0	57.4	44.3
	2005–2007	65.2	62.4	62.3	59.5	49.7	68.1	64.2	63.5	59.2	46.1
Spain[23]	1995–1999	63.9	61.1	57.3	56.1	47.2	61.0	55.4	55.6	51.0	39.0
Estonia[32]	1995–1999	60.0		47.0	52.0	44.0	41.0		43.0	44.0	26.0
	2000–2004	51.0		51.0	51.0	45.0	43.0		53.0	54.0	34.0
	2005–2009	58.0		57.0	53.0	50.0*	59.0		58.0	53.0	44.0*
	2010–2014	72.0		62.0	58.0	49.0	66.0		59.0	57.0	47.0
Africa											
Uganda											
Kampala[18]	1993–1997	0.0	13.1	0.0	18.7	0.0	–	40.5	0.0	0.0	0.0

*Excluded patients aged exceeding 85 in the original article.

Table S6 Population-based age-specific 5-year relative survival rates of colorectal cancer during 1990–2010

Regions	Period	Colorectal					
		≤ 34	35–44	45–54	55–64	65–74	≥ 75
Asia							
China							
Zhejiang[40]	2005–2010	58.9	63.1	63.5	66.0	59.0	47.4
Qidong, Jiangsu[39,43]	1972–2011	33.4	38.0	41.7	41.8	32.7	25.4
	2001–2007	42.2	41.1	50.0	54.4	50.1	42.4
Japan[35]	1993–1996	67.0		68.3	69.8	70.5	62.7
Korea[37]	2006–2010	–	–	–	–	72.3	52.5
North America							
USA[34]	2002–2006	67.8					59.8
Canada[10]	2006–2008	67.4			68.6	66.9	58.2

Table S6 Continued

Regions	Period	Colorectal					
		≤ 34	35–44	45–54	55–64	65–74	≥ 75
Europe							
Europe[45,–46]	1990–1994	57.0		55.0	53.0	51.0	43.0
	1995–1999	61.9		59.3	60.1	55.6	47.1
Germany[13,34]	2002	64.0		63.8	63.3	60.6	56.4
	2002–2006	65.4					57.2
	2006	72.0		66.4	68.9	66.0	58.8

–The age group did not match the original article.
